# Correlation between the Closure Time of Patent Ductus Arteriosus in Preterm Infants and Long-Term Neurodevelopmental Outcome

**DOI:** 10.3390/jcdd11010026

**Published:** 2024-01-16

**Authors:** Natsumi Kikuchi, Taichiro Goto, Nobuyuki Katsumata, Yasushi Murakami, Tamao Shinohara, Yuki Maebayashi, Aiko Sakakibara, Chisato Saito, Yohei Hasebe, Minako Hoshiai, Atsushi Nemoto, Atsushi Naito

**Affiliations:** 1 Department of Neonatology, Perinatal Center, Yamanashi Prefectural Central Hospital, Kofu 400-8506, Japan; k-natsumi@yamanashi.ac.jp (N.K.); katsumata-bdvt@ych.pref.yamanshi.jp (N.K.); murakami-bfwf@ych.pref.yamanashi.jp (Y.M.); t-shinohara1801@ych.pref.yamanashi.jp (T.S.); maebayashi-bfxc@ych.pref.yamanashi.jp (Y.M.); a-sakakibara1650@ych.pref.yamanashi.jp (A.S.); c-saitou1667@ych.pref.yamanashi.jp (C.S.); a-nemoto@ych.pref.yamanashi.jp (A.N.); naitou-amse@ych.pref.yamanashi.jp (A.N.); 2 Department of Pediatrics, School of Medicine, Yamanashi University, Kofu 409-3821, Japan; hasebe@yamanashi.ac.jp; 3 Lung Cancer and Respiratory Disease Center, Yamanashi Prefectural Central Hospital, Kofu 400-8506, Japan; 4 Cardiovascular Center, Yamanashi Prefectural Central Hospital, Kofu 400-8506, Japan; hoshiai-bcwe@ych.pref.yamanashi.jp

**Keywords:** patent ductus arteriosus (PDA), preterm infant, neurodevelopment, surgical ligation

## Abstract

In patent ductus arteriosus (PDA) in preterm infants, the relationship between treatment timing and long-term developmental prognosis remains unclear. The purpose of this study was to clarify the relationship between the age in days when ductus arteriosus closure occurred and long-term development. Preterm infants with a birth weight of less than 1500 g who were admitted to our NICU over a period of 9 years (2011–2019) and were diagnosed with PDA were included. A new version of the K-type developmental test for corrected ages of 1.5 and 3 years was used as an index of development. The relationship between the duration of PDA and the developmental index was evaluated using Pearson’s correlation coefficient, and multiple regression analysis was performed. Development quotient (DQ) at the ages of 1.5 and 3 years showed a correlation with the PDA closure date and the standard deviation (SD) value of the term birth weight. Multiple regression analysis showed a positive correlation of the DQ at 1.5 and 3 years with the SD value of the term birth weight and a negative correlation with the PDA closure date. In addition, a stronger correlation was found in the “posture/motor” sub-item at 3 years. On the other hand, the analysis including preterm infants without PDA showed that preterm infants with PDA closure on the 6th day or later after birth had a significantly lower 3-year-old DQ than preterm infants with a PDA exposure within 5 days. In conclusion, it is suggested that the decrease in cerebral blood flow due to PDA in preterm infants has an adverse effect on long-term neurodevelopment. Appropriate interventions, including surgical treatment for PDA in preterm infants without delay, ideally within 5 days of birth, may be effective in improving the developmental prognosis.

## 1. Introduction

The ductus arteriosus is a blood vessel that connects the aorta and the pulmonary artery during the fetal period and closes spontaneously 12 to 48 h after birth [1]. During fetal circulation, the ductus arteriosus directs blood flow from the fetal pulmonary artery to the aorta and plays an important role in ensuring oxygen supply during periods when the fetal lungs are immature and lung function is limited [2]. At birth, a decrease in prostaglandin E2 levels, an increase in arterial oxygenation, and a decrease in pulmonary vascular resistance promote the contraction of the ductus arteriosus, forming a fibrous tissue [2]. The condition in which the ductus arteriosus remains patent after 72 h postpartum is called patent ductus arteriosus (PDA) in preterm infants. The lower the gestational age and the lower the birth weight, the higher the frequency of onset of the disease [3]. PDA is diagnosed in 39% of very-low-birth-weight infants (VLBW, <1500 g birth weight), and up to 70% of infants born before 28 wk gestation, and there have been many cases of long-term patent ductus arteriosus in preterm infants [4]. PDA is associated with neonatal mortality and morbidity, including conditions such as bronchopulmonary dysplasia (BPD), necrotizing enterocolitis (NEC), and intraventricular hemorrhage (IVH) [5,6,7,8]. In addition, there are many unclear points regarding its impact on long-term neurodevelopment. In many countries, the treatment of PDA in preterm infants is initiated only after symptoms become evident [9], while in Japan, it is diagnosed prior to symptom manifestation by frequent echography, and thus, the treatment is started earlier [10]. There is no sufficient scientific basis for the treatment intervention criteria for PDA in preterm infants, and the current situation is that the diagnosis and the treatment based on clinicians’ experience are carried out independently in each country and institution. In Japan, if the ductus arteriosus does not close spontaneously, COX inhibitors (e.g., indomethacin) are administered according to the established guidelines [10,11,12]. At our hospital, if closure is not achieved after the first course of administration, a second course of administration is carried out, and then, surgical treatment is performed in those cases in which closure has still not been achieved. However, in such cases, most infants underwent surgical intervention 1 month after birth.

There is still no consensus on when it is best to close a PDA in the circulatory management of preterm infants [13,14,15], and various treatments are provided depending on the custom at each institution, ranging from prophylactic treatment to conservative treatment. There is an urgent need to determine the optimal timing of treatment for PDA, but to date, there have been no reports on the relationship between PDA closure time and long-term development, and therefore, no unambiguously clear treatment guideline has been established. Thus, this study was undertaken for the purpose of evaluating the risk of neurodevelopmental impairment in preterm infants with PDA.

## 2. Methods

### 2.1. Patients

Infants who were admitted to our NICU from January 2011 to December 2019, had a gestational age of less than 32 weeks or a birth weight of less than 1500 g, and required treatment for PDA (in preterm infants) were included in our study. Cases in which a developmental evaluation was not achieved due to chromosomal abnormalities and death/relocation were excluded. This study was conducted in compliance with the ethical principles in the WMA Declaration of Helsinki and approved by the Institutional Review Board Committee of Yamanashi Central Hospital (protocol code: Clin20-66). From the NICU database and outpatient medical records, items of terms of age in days when ductus arteriosus closure occurred (PDA closure date), birth weight, the SD value of the body weight on the date of term birth or discharge (SD value of term birth weight), age when nutrition reached 100 mL/kg, and the presence of any complications (chronic lung disease, intraventricular hemorrhage, periventricular leukomalacia, sepsis, and/or idiopathic gastrointestinal perforation) were extracted. Of the infants hospitalized during the above study period, developmental tests were performed on infants who attained the age of one and a half and three years. At the corrected ages of 1.5 and 3 years, the developmental quotient (DQ) value was calculated on the new version of the K-type developmental test, and the respective sub-items (posture/motor, cognition/adaptation, and language/sociality) were also examined.

### 2.2. Statistical Analysis

Continuous variables were presented as the mean ± standard deviation (SD) and were compared using unpaired Student’s *t*-tests. Chi-square tests were used to compare the categorical data between groups. Correlation analysis was tested using Pearson’s correlation coefficient. Subsequently, the items for which a significant difference was found were further subjected to multivariate analysis using multiple regression analysis (stepwise method). *p*-values of less than 0.05 in the two-tailed analyses were considered to denote statistical significance.

## 3. Results

### 3.1. Patient Characteristics

Preterm infants admitted to our NICU from 2011 to 2019 were included in this study. Of the infants with a gestational age of less than 32 weeks or a birth weight of less than 1500 g, 114 cases had PDA. Of the preterm infants admitted to our NICU at the same time, 155 cases did not have PDA and were included in the control group. Upon comparing preterm infants with and without PDA, the birth weight and the SD value of term birth weight were significantly lower, and the duration of enteral nutrition was significantly longer in the PDA+ group (Table 1). In addition, the proportion of infants with chronic lung disease and intraventricular hemorrhage was significantly higher in the PDA+ than that in the PDA− group.

The mean age in days when ductus arteriosus closure occurred in 114 cases with PDA was 10.1 days old. When classified into three groups (spontaneous closure, COX inhibitor, and surgery) based on the PDA closure mode, the spontaneous closure group included 155 cases (57.6%), the COX inhibitor group included 97 cases (36.1%) with a mean age in days when ductus arteriosus closure occurred of 11.5 ± 20.0 days, and the surgery group included 17 cases (6.3%) with a mean age in days when ductus arteriosus closure occurred of 11.2 ± 17.7 days.

### 3.2. Developmental Test Results

**Figure 1:**
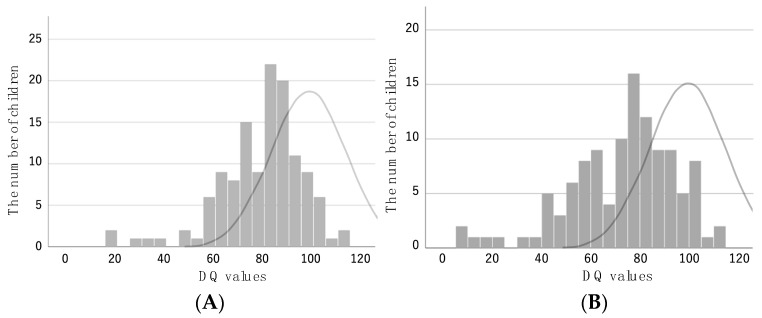
Distribution of DQ values at the ages of 1.5 (**A**) and 3 years (**B**) calculated on the new version of K-type developmental test. The curve shows the distribution of the data for all Japanese infants of the same age.

Preterm infants with PDA had significantly lower 3-year-old DQ values than those without PDA (PDA present: 73.4 ± 21.5; PDA absent: 84.6 ± 15.5; *p* < 0.05). The results of the developmental tests of 126 and 114 preterm infants with PDA at ages of 1.5 and 3 years, respectively, were distributed as shown in Figure 1.

### 3.3. Analysis of the Factors That Influenced the Developmental Test Results

Univariate analysis showed that the 1.5-year-old DQ values significantly correlated with the birth weight, the PDA closure date, the SD value of the term birth weight, and periventricular leukomalacia. Multivariate analysis showed that they significantly correlated with the PDA closure date, the SD value of the term birth weight, chronic lung disease, and periventricular leukomalacia (Table 2). Univariate analysis showed that the 3-year-old DQ value significantly correlated with the birth weight, the PDA closure date, the SD value of the term birth weight, and chronic lung disease. Multivariate analysis showed they significantly correlated with the PDA closure date and the SD value of the term birth weight (Table 3). When the relationship between the sub-items of the 3-year-old DQ value (posture/motor, cognition/adaptation, and language/sociality) and the PDA closure date were examined, the PDA closure date was found to significantly correlate with posture/motor and cognition/adaptation as well as posture/motor with univariate and multivariate analyses, respectively (Table 4).

### 3.4. Comparison of Preterm Infants with and without PDA

**Figure 2:**
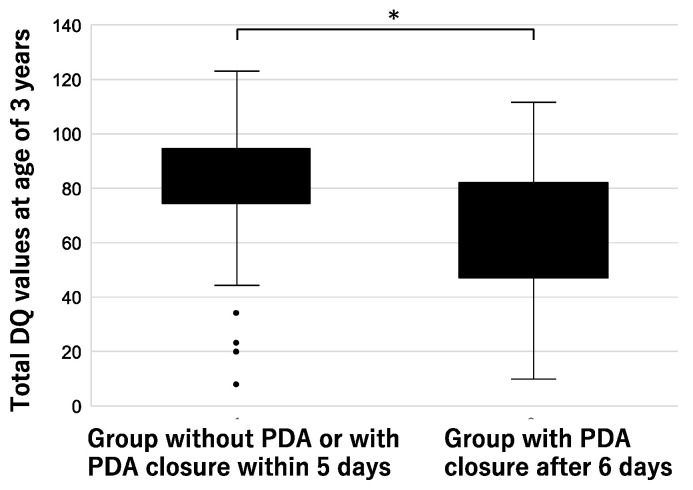
Changes in 3-year-old DQ values according to duration of PDA. *, *p* < 0.05.

When preterm infants without PDA were regarded as PDA exposure time = day 0, and the correlation with the 3-year-old DQ value was examined with multivariate analysis, and a significant correlation was found with the birth weight, the PDA closure date and chronic lung disease (Table 5). When ROC analysis was used to examine the cut-off value of the PDA exposure time, which specified the 3-year-old DQ value, the maximum value of AUC (=0.70) was achieved with PDA closure on day 5. If the PDA is closed within 5 days, the DQ is equivalent to those without PDA, and the group without PDA or with PDA closure within 5 days had a significantly higher 3-year-old DQ value than the group with PDA closure after 6 days (Figure 2, *p* < 0.05).

## 4. Discussion

In this study, we examined the correlation between the age in days when ductus arteriosus closure occurred and neurodevelopment at the ages of 1.5 and 3 years in preterm infants with PDA and found that the DQ values at the ages of 1.5 and 3 years negatively correlated with the PDA closure date. In particular, a stronger correlation was found for the sub-item “posture/motor” of the 3-year-old DQ. On the other hand, the analysis including preterm infants without PDA showed that preterm infants with PDA closure at 6 days or longer after birth had a significant decrease in the 3-year-old DQ compared with that of preterm infants with PDA closure within 5 days.

The ductus arteriosus is the vehicle for the normal traffic that connects the pulmonary artery and the aorta and is necessary for proper fetal circulation [16]. Elevated PaO_2_ (blood oxygen levels) and decreased prostaglandin levels at birth lead to the initiation of ductus arteriosus closure, typically within 10 to 15 h after birth [17]. If this process is not successful, the ductus arteriosus will remain patent. Ductus arteriosus closure is delayed in the premature state, since it is not yet programmed for prompt postnatal closure. Especially when PDA closure is not achieved in preterm infants, a decrease in body blood flow and an increase in pulmonary blood flow might occur with the underdevelopment of heart function, resulting in brain disorders such as intraventricular hemorrhage, renal disorders, necrotizing enterocolitis, and pulmonary hemorrhage, as well as acute symptoms such as heart failure [18,19,20,21].

This study consecutively collected cases in a single institution, with slight difference in the ratio of the closure mode of the ductus arteriosus detected between the current study (57.6%, 36.1%, and 6.3% of subjects in the spontaneous closure, COX inhibitor, and surgical intervention groups, respectively) and other clinical studies with extremely low birth weight (ELBW) infants reporting 14 to 39%, 42 to 63%, and 23 to 32%, respectively [22,23,24,25]. Recently, conservative therapy has become more common, and the proportion of medical and surgical management has tended to decrease [26]. Thus, the measurement bias associated with the case bias is judged to be small in this study. In addition, a delayed closure of the ductus arteriosus was associated with a decrease in DQ values at the ages of 1.5 and 3 years, especially the decrease in the motor area. Furthermore, multiple regression analysis showed that delayed PDA closure correlated with delayed neurodevelopment independent of the SD value of the term birth weight. It has been assumed that PDA in preterm infants may affect long-term neurodevelopment through certain mechanisms such as decreased cerebral blood flow and deteriorated nutrition, but our data suggest that only the decrease in cerebral blood flow due to PDA, and not the “prematurity-related deterioration of nutritional status” directly leads to the decrease in neurodevelopment. Cooke et al. discussed the notion that PDA-mediated shunts from systemic flow to pulmonary flow and hypotension and decreased perfusion and oxygenation of the preterm brain caused by immature myocardium might affect brain development and negatively affect neurodevelopment outcomes [27]. Moreover, it is reported that long-term exposure to PDA in preterm infants decreased the oxygen saturation in cerebral tissue and resulted in a volume reduction of whole brain regions at term age [28]. In this study, it was presumed that PDA prolongation adversely affected long-term neurodevelopment through the same mechanism. On the other hand, there are some reports that chronic lung disease affects subsequent growth and development [29,30,31], but in our study, there was no correlation between the covariable of chronic lung disease and neurodevelopment at the age of 3 years, suggesting persistent PDA is more involved in neurodevelopment impairment than chronic lung disease.

In the treatment of PDA, many asymptomatic cases are often followed up with water restriction alone. The main reason for applying a conservative approach is that spontaneous permanent DA closure occurs in >34% of ELBW neonates [32]. In preterm infants with the deterioration of respiratory status due to hemodynamically significant PDA, ductus arteriosus closure might be attempted through prostaglandin production inhibition by the administration of COX inhibitors (ibuprofen or indomethacin) [33]. In addition, the Japanese guidelines recommend surgical ligation for PDA upon unsuccessful water restriction and/or administration of COX inhibitors [10,12]. However, surgical treatment after the combined use of COX inhibitors delays the treatment timing by about 1 to 2 months, and it is also reported that the repeated administration of COX inhibitors may inhibit intimal thickening in the ductus arteriosus and thereby delay closure [34], likely resulting in an increase in the PDA exposure time [22,24,35]. Thus, the optimal timing of ductus arteriosus closure remains controversial, and there is even the view that patent ductus arteriosus is not a treatment target in preterm infants who does not present respiratory problems or other abnormalities [36]. However, the results of this study do not agree with this view. This study showed that the delayed closure of PDA adversely affects neurodevelopment, suggesting that it is necessary to select the treatment strategy with immediate closure in mind.

In this context, the incidence of neurodevelopmental impairment in ELBW infants might be improved by the clarification of the optimal timing for surgical treatment and the consequent shortening of the exposure time to PDA. Although this study concluded that PDA closure within 5 days was advantageous for the long-term outcomes of neurodevelopment, surgical treatment immediately after birth may be overly invasive. It has been reported that ligation in the early postnatal period (especially within 10 days of age) may adversely affect the prognosis for neurodevelopment [21,35], and the cause is presumed to be due to the level of maturity of the infant at the time of surgery. Sung et al. showed that delayed ligation (after more than 2 weeks after birth) decreased the risk for mortality or morbidities in extremely preterm infants born at 23–25 weeks of gestation [37]. Therefore, as a method that can be performed immediately after the birth of preterm infants and is minimally invasive, the transcatheter closure of PDA for preterm infants weighing less than 2 kg has been reportedly successful in a small number of institutions [38,39,40]. Considering the optimization of outcomes for long-term neurodevelopment, we hope that new, feasible, and effective treatments for this PDA will become widespread in the future.

In Japan, the Piccolo Occluder became commercially available in April 2020, allowing the transcatheter treatment of underweight infants. The device is made of woven nickel-titanium alloy (Nitinol) wires and can be implanted via 4 French catheters and can be used in children weighing 700 g or more. Transcatheter closure remains the standard of care in children and infants weighing >6 kg. A multicenter retrospective study by Dimas et al. analyzed the results of transcatheter PDA closure in infants weighing <6 kg [41]. The study concluded that transcatheter PDA closure is effective and safe in infants weighing 6 kg or less. However, data on the outcome of transcatheter PDA closure in very preterm infants are inconclusive [42].

Although there are reports that catheterization with the Piccolo Occluder is performed at the bedside of the NICU with echo-guidance or mobile fluoroscopic guidance [43,44], most of them are performed by transferring the patient to the catheterization room. In such cases, careful preparation is necessary, such as moving the patient, keeping the catheterization room warm, and setting up a single ventilator. Because this procedure is currently available only at a limited number of accredited facilities, it is necessary to prepare simulations in advance through the local NICU network because it is expected to be performed between local facilities. Sathanandam et al. reported that the frequency of complications was significantly lower with transcatheter closure, at 3.3% and 25.7%, respectively [45]. In 241 transcatheter procedures in patients weighing 500–2000 g, one procedure-related death, one retrieval due to device failure, and one retrieval due to left pulmonary artery stenosis were reported, and in 167 surgical procedures, two procedure-related deaths, wound dehiscence, residual short circuit requiring re-intervention, pneumothorax, sepsis, decreased cardiac function, the deterioration of respiratory status, and post-ligation syndrome were reported [46]. In a trial using the Piccolo Occluder, the success rate was 99% (99/100) in patients weighing over 700 g and 95.5% (191/200) in patients weighing less than 5 kg at the time of implantation [40]. Two cases of transfusion, one case of aortic stenosis, and five cases of worsening tricuspid regurgitation were observed, but no bifurcation pulmonary artery stenosis was observed, proving the high efficacy and safety of the procedure. An important complication of surgery is known as post-ligation syndrome, which is caused by an increase in afterload due to the sudden blockage of the ductus arteriosus and a decrease in left ventricular preload, resulting in a decrease in cardiac output. On the other hand, this syndrome is extremely rare in transcatheter closure procedures [47].

A recent meta-analysis evaluated the safety and efficacy of transcatheter closure compared to surgical ligation in 756 preterm infants with PDAs weighing less than 2000 g birth weight [48]. Compared to transcatheter closure, surgical ligation had a higher mortality rate. On the other hand, there were no significant differences in post-procedure complication rates, the mean duration of mechanical ventilation after the procedure, the length of hospital stay, or the length of stay in the neonatal intensive care unit. In addition, a subgroup analysis of the Regan et al. cohort showed that infants younger than 4 weeks of age who underwent transcatheter closure had a shorter hospital stay compared to surgical ligation [49]. The advantages of the transcatheter procedure include no larger incision, minimally invasive, and the low risks of infection, bleeding, scar formation, and chest wall deformity [50]. Disadvantages include the limited number of skilled interventional cardiologists, the limited number of centers where treatment is available, and the lack of data on long-term outcomes, which require further analysis based on case series [51]. With the recent improvements in devices, the accreditation of facilities and interventional cardiologists, and the spread of safe procedures, the option of catheterization within 5 days after birth, as suggested in this study, could become the standard of care in the near future. On the other hand, not all cases of PDA can be treated with transcatheter closure, and surgical closure remains a viable option for infants with complex anatomy or significant complications. Therefore, an in-depth case review by a multidisciplinary team of cardiologists, neonatologists, and pediatric cardiac surgeons is needed to make informed decisions.

The limitations of this study include the fact that it is a single-center study, the sample size is small, there are biases associated with the retrospective design, and the severity of the PDA itself may reflect the severity of the preterm infants’ condition that would thus be a confounding factor [51,52]. However, in this study, in order to eliminate the possibility of such a conflicting factor, the birth weight was also added to the analysis as a covariate and as an index reflecting immaturity, and the results turned out to be not influenced by bias related to birth weight. In addition, the correlation coefficient between the covariables of birth weight and the PDA closure date was approximately 0.4, and each index was judged to be an independent factor. Multivariate analyzes involving a large number of patients and multicenter prospective clinical studies need to be conducted to further determine the critical time of exposure to PDA in ELBW infants. In addition, there are still many unclear points regarding the mechanisms by which delayed PDA closure affects development, and further elucidation is both needed and expected. This study aimed to correct some of the inadequacy of the PDA treatment policy, and we believe that even a modestly sized sample such as this one is able to provide useful insight for this purpose.

## 5. Conclusions

Prolonged PDA may increase the risk of long-term neurodevelopmental impairment. Appropriate interventions, including surgical treatment (surgery and catheterization) for patent ductus arteriosus in preterm infants without delay, ideally within 5 days of birth, are suggested to be effective in improving the developmental prognosis.

## Figures and Tables

**Table 1:** Patients’ characteristics.

	PDA Present	PDA Absent	*p* Value
Number of patients	114	155	
Male/female	47/67	77/78	0.17
Birth weight (g)	899 (376–1496)	1232 (496–1802)	<0.01
SD value of term birth weight	−3.28 (−15.3–0.31)	−2.56 (−17.5–1.56)	<0.05
Enteral nutrition (day)	17.7 (8–60)	9.8 (1–48)	<0.05
Chronic lung disease (%)	70 (61.4)	25 (16.1)	<0.01
Intraventricular hemorrhage (%)	13 (11.4)	1 (0.65)	<0.01
Periventricular leukomalacia (%)	5 (4.4)	1 (0.65)	0.10
Sepsis (%)	10 (8.8)	9 (5.8)	0.49
Idiopathic gastrointestinal perforation (%)	1 (0.9)	1 (0.65)	0.62

**Table 2:** Correlation with the 1.5-year-old DQ value.

	Univariate Analysis		Multivariate Analysis	
	Pearson’s Correlation Coefficient	*p* Value	*t* Value	*p* Value
Birth weight	0.220	0.013	0.826	0.410
PDA closure date	−0.241	0.006	−2.451	0.016
SD value of term birth weight	0.347	<0.001	2.480	0.015
Chronic lung disease	−0.235	0.080	2.371	0.019
Periventricular leukomalacia	−0.292	0.001	3.431	0.001

**Table 3:** Correlation with the 3-year-old DQ value.

	Univariate Analysis		Multivariate Analysis	
	Pearson’s Correlation Coefficient	*p* Value	*t* Value	*p* Value
Birth weight	0.218	0.020	0.661	0.510
PDA closure date	−0.234	0.012	−2.034	0.044
SD value of term birth weight	0.399	<0.001	4.285	<0.001
Chronic lung disease	−0.206	0.028	1.026	0.307
Periventricular leukomalacia	−0.163	0.083	1.175	0.242

**Table 4:** Correlation between the PDA closure date and the sub-items of the developmental test at age of 3 years.

	Univariate Analysis		Multivariate Analysis	
Dependent Variables	Pearson’s Correlation Coefficient	*p* Value	*t* Value	*p* Value
Posture/motor	−0.312	0.001	−3.444	0.001
Cognition/adaptation	−0.208	0.027	−0.651	0.517
Language/sociality	−0.132	0.163	−0.158	0.874

**Table 5:** Correlation between PDA exposure time and the 3-year-old DQ value, including infants without PDA.

	Univariate Analysis		Multivariate Analysis	
	Pearson’s Correlation Coefficient	*p* Value	*t* Value	*p* Value
Birth weight	0.375	<0.001	2.163	0.031
PDA exposure time	−0.273	<0.001	−2.866	0.004
SD value of term birth weight	0.277	<0.001	2.562	0.011
Chronic lung disease	0.355	<0.001	2.131	0.034

## Data Availability

The data presented in this study are available on request from the corresponding author. The data are not publicly available due to them containing information that could compromise research participant privacy/consent.
